# Hebbian activity-dependent plasticity in white matter

**DOI:** 10.1016/j.celrep.2022.110951

**Published:** 2022-06-14

**Authors:** Alberto Lazari, Piergiorgio Salvan, Michiel Cottaar, Daniel Papp, Matthew F.S. Rushworth, Heidi Johansen-Berg

**Affiliations:** 1 Wellcome Centre for Integrative Neuroimaging, FMRIB, Nuffield Department of Clinical Neurosciences, University of Oxford, Oxford OX2 6GG, UK; 2 Wellcome Centre for Integrative Neuroimaging, Department of Experimental Psychology, University of Oxford, Oxford OX2 6GG, UK

**Keywords:** myelin, myelin plasticity, brain plasticity, brain stimulation, magnetic resonance imaging, white matter, Hebbian plasticity, action reprogramming

## Abstract

Synaptic plasticity is required for learning and follows Hebb’s rule, the computational principle underpinning associative learning. In recent years, a complementary type of brain plasticity has been identified in myelinated axons, which make up the majority of brain’s white matter. Like synaptic plasticity, myelin plasticity is required for learning, but it is unclear whether it is Hebbian or whether it follows different rules. Here, we provide evidence that white matter plasticity operates following Hebb’s rule in humans. Across two experiments, we find that co-stimulating cortical areas to induce Hebbian plasticity leads to relative increases in cortical excitability and associated increases in a myelin marker within the stimulated fiber bundle. We conclude that Hebbian plasticity extends beyond synaptic changes and can be observed in human white matter fibers.

## Highlights

• Induction of Hebbian plasticity in human brain using non-invasive Hebbian stimulation
• Hebbian plasticity induction leads to changes within the stimulated fiber bundle
• Anatomically relevant behavioral changes in action reprogramming
• Activity-dependent white matter plasticity operates following Hebb’s rule

## Graphical abstract

**Figure:**
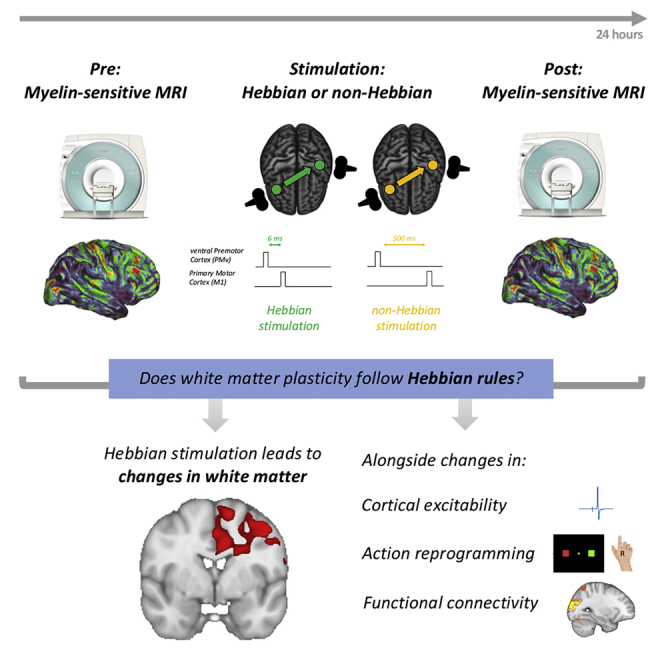

## Teaser

Lazari et al. demonstrate that Hebbian activity-dependent plasticity extends beyond synaptic changes and can be observed in human white-matter fibers.

## Introduction

Hebb’s rule (Hebb, 1949) has been extremely influential in neuroscience. It postulated for the first time that a computational principle could link a biological process (“neurons that fire together, wire together”) with a cognitive process (Pavlovian/associative learning), an idea that has become pivotal for neuroscience research. Hebb’s rule was later found to have a biological substrate in the synapse. Synapses can detect coincident activity of two neurons, i.e., detect when neurons “fire together,” and effect plastic changes in the synaptic connections between them, i.e., make neurons “wire together” (Bliss and Lømo, 1973). Strikingly, more than half a century after it was first proposed, Hebbian theory is still thought to be accurate, although it is now encompassed by wider frameworks such as spike-timing-dependent plasticity (Bi and Poo, 1998) or Bienenstock-Cooper-Munro theory (Bienenstock et al., 1982). In addition, extensive evidence has demonstrated that synaptic plasticity and its Hebbian properties are crucial for learning (Tsien et al., 1996; Ryan et al., 2015).

In recent years, another key site of brain plasticity has been identified: the myelinated axon (Almeida and Lyons, 2017). Myelinated axons make up the majority of brain’s white matter, where this form of plasticity was first identified in humans (Scholz et al., 2009). This distinct plastic process has been confirmed to have two properties similar to synaptic plasticity: it is activity dependent, and it is implicated in learning. Its activity dependence has now been confirmed in animal models across a broad range of methods, including electrical stimulation (Li et al., 2010), optogenetics (Gibson et al., 2014), chemogenetics (Mitew et al., 2018), prevention of synaptic vesicle release by tetanus toxin (Mensch et al., 2015), and non-invasive transcranial magnetic stimulation (Cullen et al., 2021). Regarding its link to behavior, active myelination is critical for a wide range of learning behaviors (Kaller et al., 2017), including motor learning (McKenzie et al., 2014), fear learning (Pan et al., 2020), and spatial memory (Steadman et al., 2019).

However, unlike synaptic plasticity, myelin plasticity has not been directly linked to known computational principles, and it is still unclear what rules it might follow. Our study was designed to test whether myelin plasticity follows Hebb’s rule. To induce short-term plasticity, we used non-invasive transcranial magnetic stimulation (TMS) to elicit neuronal activity with tight temporal control over two brain areas in a Hebbian fashion (Buch et al., 2011; Johnen et al., 2015). We then combined Hebbian stimulation with magnetic resonance (MR)-based quantitative myelin markers to detect myelin changes induced by Hebbian stimulation.

To facilitate a biological interpretation of our results, we focused on magnetization transfer saturation (MT), an MR-based metric that has been extensively validated with myelin histology (Lazari and Lipp, 2021; Mancini et al., 2020). Unlike in rodent experiments, in which it is possible to carefully control the environments of experimental subjects, the possibility of variation in environment across human participants meant that care had to be taken to ensure that measures of physiology and myelination would have the best chance of revealing any impact of plasticity that might have occurred. We therefore scanned participants 24 h before and after Hebbian stimulation. This time frame was selected to be sensitive to both the physiological changes that are associated with Hebbian plasticity (which are apparent soon after Hebbian stimulation [Buch et al., 2011]) and myelination-related effects, such as remodeling of myelin morphology, changes in the length of nodes of Ranvier, or production of myelin from existing oligodendrocytes (Yeung et al., 2014; Young et al., 2013; Arancibia-Carcamo et al., 2017; Bacmeister et al., 2020). These myelination-related effects may take slightly longer but can occur within 24 h (Almeida and Lyons, 2017; Yeung et al., 2014; Young et al., 2013; Arancibia-Carcamo et al., 2017; Bacmeister et al., 2020) and are all known to impact MT measurements (Lazari and Lipp, 2021; Mancini et al., 2020).

## Results

### Inducing Hebbian plasticity in the human brain

**Figure 1:**
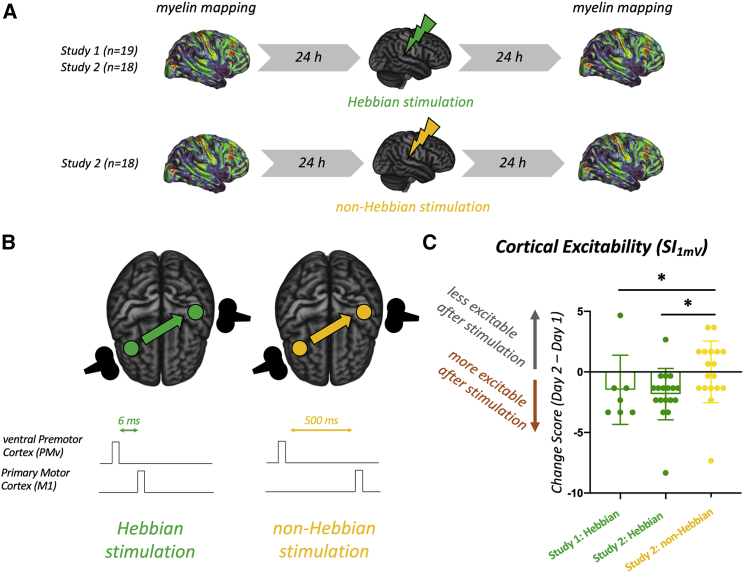
Inducing Hebbian plasticity in the human brain (A) Summary of experimental design, using two cohorts to establish effects of Hebbian stimulation on brain microstructure. Study 1 (n = 19) included the Hebbian condition only. In study 2, a different set of individuals were randomized to receive either Hebbian (n = 18) or non-Hebbian (n = 18) stimulation. (B) Diagram of the Hebbian (active) and non-Hebbian (control) conditions used in the experiments. Both stimulation protocols are matched for duration, intensity, and coil location but differ in the relative timing of the stimulation pulses, with the Hebbian condition aiming to mimic the timing of synaptic plasticity inductions used *in vitro*. (C) Longitudinal effects of Hebbian-plasticity induction on cortical physiology. Each dot in the graph represents the normalized change in cortical excitability (as measured by the SI_1mV_ metric) for one subject. The SI_1mV_ measure was collected in an exploratory manner in the last 7 participants of study 1 and in all participants of study 2 to confirm the presence of longitudinal effects.

We used Hebbian stimulation in healthy adult participants to induce associative plasticity between the right ventral premotor cortex (PMv) and the left primary motor cortex (M1) (Figures 1A, 1B, S1A, and S1B; see also STAR Methods, experimental design). First (study 1: Hebbian; Figures 1A and 1B), we checked that a protocol, which has been shown to induce Hebbian plasticity (Buch et al., 2011; Johnen et al., 2015), induced a measurable change in the excitability of M1 between the two testing days; this was indeed the case (left bar, Figure 1C; subset of n = 7 participants from study 1). We then repeated the same testing protocol in a separate group of 18 participants (study 2: Hebbian) and compared it with a control procedure, which we refer to as “non-Hebbian stimulation,” conducted in another group of 18 participants (study 2: non-Hebbian). When we compared changes in excitability in left M1 between the two testing days in the two conditions in study 2, we found that there was a clear difference (Figure 1C, study 2: Hebbian versus study 2: non-Hebbian, Mann-Whitney U test, p = 0.0049). While reductions in excitability over time were observed for the control condition (as expected from similar longitudinal studies [Schlamann et al., 2010]), this effect was rescued by the Hebbian-plasticity-induction protocol, resulting in relatively greater M1 excitability following the Hebbian procedures. We then pooled together data from all participants and found again that changes in cortical excitability of left M1 differed between the stimulation conditions (one-way ANOVA, F (2, 42) = 8.747, p = 0.0126). This effect was present 24 h after stimulation, indicating a long-lasting physiological effect of the stimulation compatible with the longer timescales expected in myelin plasticity (Almeida and Lyons, 2017).

### Hebbian activity-dependent plasticity in white matter

**Figure 2:**
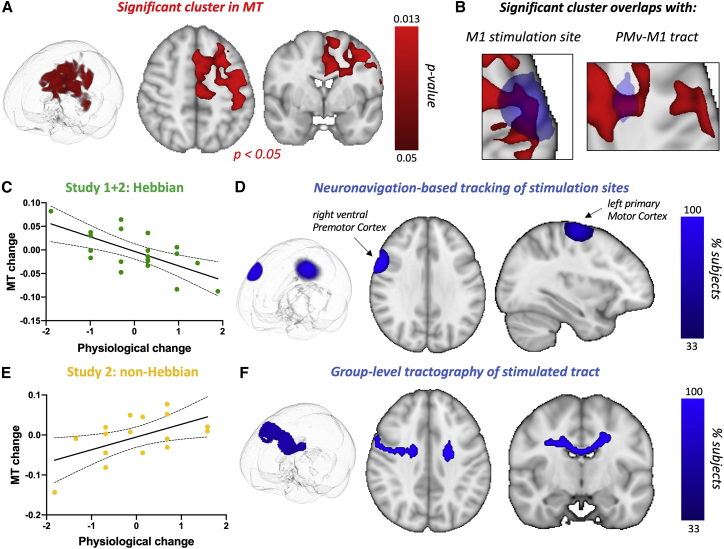
Microstructural plasticity induced by Hebbian stimulation (A) Results from a whole-brain analysis identify a cluster where changes in MT values correlate with changes in cortical excitability in the Hebbian condition significantly more than they do in the non-Hebbian condition. (B) The significant MT cluster identified by the whole-brain analysis (red) overlaps with stimulation sites in the gray matter and with the stimulated fiber tract in the white matter (blue). (C and E) Scatterplots of data underlying the significant cluster. For the Hebbian condition, participants with greater increases in excitability (more negative physiological change score) show greater increases in MT. Each data point is a single participant; scatterplots (with line of best fit and 95% confidence bands) are presented for post-hoc visualization of the correlations rather than for statistical inference. (D and F) Tracking of stimulation sites via neuronavigation allows us to estimate the location of cortical stimulation sites and to reconstruct the stimulated fiber bundle in white matter.

To test whether Hebbian stimulation induced myelin plasticity, we collected highly reliable (Figure S2A) whole-brain myelin-sensitive MT maps 24 h before and after Hebbian stimulation in study 1 and study 2. Group comparisons of changes in MT did not detect significant differences between Hebbian and non-Hebbian conditions. However, using a whole-brain analysis, we were able to test whether physiological changes induced through Hebbian stimulation were associated with changes in myelin maps anywhere in the brain. We found a significant cluster in which participants with the strongest increases in cortical excitability, specifically following Hebbian stimulation, also exhibited the strongest increases in MT (Figure 2A, peak p_corr_ = 0.013). In both studies, this effect was present in those receiving Hebbian stimulation (Figure S2B) but was not present in those receiving non-Hebbian stimulation (Figures 2C and 2E).

We investigated the anatomical relationship between Hebbian stimulation and this cluster. We found that in cortical areas, the cluster overlapped with locations of the M1 coil that were recorded during stimulation. We then performed tractography and reconstructed the stimulated white matter bundle connecting the stimulation sites (Figure S2C). The significant cluster overlapped with the reconstructed white matter bundle (Figures 2B, 2D, and 2F), further confirming the close relationship between Hebbian stimulation and observed myelin changes.

### Hebbian stimulation induces anatomically relevant changes in action reprogramming

**Figure 3:**
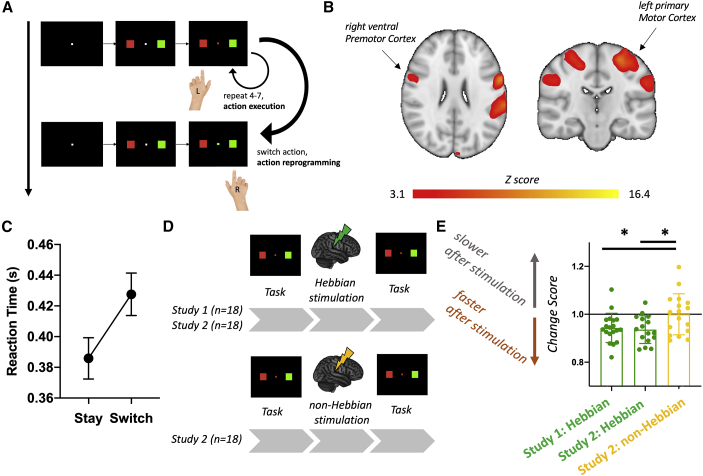
Hebbian stimulation induces anatomically relevant changes in action reprogramming (A) Schematic of the action-reprogramming task used, based on (Neubert et al., 2010), probing both action execution (stay trials) and action reprogramming (switch trials). (B) Premotor-to-motor circuits are involved in action reprogramming, as exemplified by a meta-analysis of action-reprogramming task fMRI studies. (C) Reaction times during the task increase in switch trials (when the cue changes) compared with stay trials (while the cue remains the same) in all studies. (D) Summary of experimental design, testing the effects of Hebbian stimulation on action reprogramming in two cohorts. (E) Longitudinal effects of Hebbian-plasticity induction on action-reprogramming behavior. Each dot in the graph represents the normalized change in switch-trial reaction time for one subject.

We then assessed whether the Hebbian white matter changes we observed might play a role in behaviors known to be supported by the white matter fibers being stimulated. In studies 1 and 2, subjects undertook an action-reprogramming task (Figure 3A). Action reprogramming is known to selectively involve the PMv to M1 motor circuit (Neubert et al., 2010), which we further confirmed through a meta-analysis of the action-reprogramming task functional MR imaging MRI (fMRI) literature (Figure 3B). This is consistent with the observation that not only does PMv have a major projection to M1 (Dum and Strick, 2005), which enables it to exert a strong influence over M1 activity (Davare et al., 2009; Prabhu et al., 2009) but, in addition, PMv receives an especially strong projection from lateral prefrontal cortex (Dum and Strick, 2005). It is also important to note that many PMv projections to M1 terminate on inhibitory interneurons (Tokuno and Nambu, 2000; Prabhu et al., 2009). Thus, in conjunction, PMv’s pattern of anatomical connections ensures that during action reprogramming, it can mediate inhibitory influences, originating from executive control processes in prefrontal cortex, over motor processes in M1.

We found that changes in performance on the action-reprogramming task differed between the stimulation conditions, specifically on the action-reprogramming trials of the task (but not on trials in which participants did not reprogram actions and simply made the actions that they had preprepared; Figures 3C–3E, one-way ANOVA effect of group: F (2, 51) = 4.377, p = 0.0178). While slower reaction times were observed following the control condition, this effect was rescued by the Hebbian-plasticity-induction protocol, resulting in relatively improved action reprogramming performance following Hebbian stimulation (post-hoc study 2: Hebbian versus study 2: non-Hebbian p = 0.0280; post-hoc post-hoc study 1: Hebbian versus study 2: non-Hebbian, p = 0.0447). No changes, however, were found when no action reprogramming was required and participants simply made the movements that they had preprepared (i.e., stay trials; Figure S3). Behavioral changes in action reprogramming were present even when covarying for changes in action execution performance during stay trials (one-way ANCOVA effect of group: F(2, 51) = 4.373, p = 0.018), which further supports a close link between Hebbian stimulation and the observed myelin changes.

### Functional neuroimaging reveals compensatory connectivity changes induced by Hebbian stimulation

**Figure 4:**
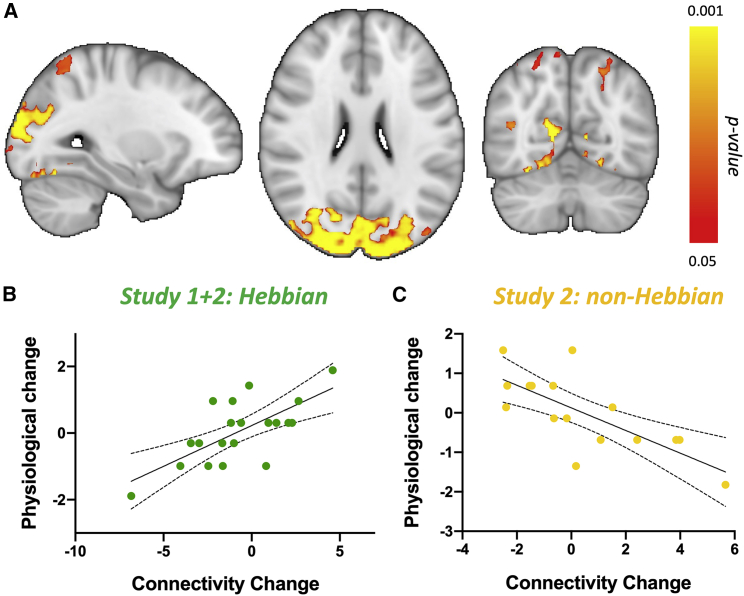
Large-scale compensatory changes in resting-state connectivity induced by Hebbian stimulation (A) Results from a whole-brain analysis identify clusters where connectivity changes correlate with changes in cortical excitability in the Hebbian condition significantly more than they do in the non-Hebbian condition. (B and C) Scatterplots of data underlying the significant cluster. Each data point is a single participant; scatterplots (with line of best fit and 95% confidence bands) are presented for post-hoc visualization of the correlations rather than for statistical inference.

Finally, it is possible that Hebbian plasticity may also induce compensatory functional changes (Johnen et al., 2015). Therefore, we tested whether Hebbian stimulation induces large-scale changes in functional connectivity of the stimulated areas. We found evidence for large-scale compensatory changes in functional connectivity (Figure 4, peak p_corr_ = 0.001). More specifically, we found that participants with the strongest increase in cortical excitability following Hebbian stimulation also exhibited the strongest decrease in connectivity between stimulated brain areas and non-stimulated visuomotor pathways (Figures 4A and 4B), including posterior superior parietal cortex (pSPL) and area V3A (Figure S4). This correlation was not present in those receiving non-Hebbian stimulation (Figure 4C).

## Discussion

Hebb’s rule provides a rare conceptual link between cellular plasticity (neurons that fire together, wire together) and cognition (associative learning) and has thus been central to how we conceive of brain function and learning. While synaptic plasticity has often been assumed to be the cellular basis for Hebbian plasticity (Tsien et al., 1996; Bannerman et al., 2014), here, we show that Hebb’s rule extends beyond synaptic changes. The neurons that fire together, wire together principle applies not only to synapses but also to myelinated long-range connections between neurons in the white matter.

As Hebbian plasticity requires the detection of coincident neuronal activity, one key implication of our findings is that plasticity in myelinated white matter tracts can be influenced by coincident activity in the areas they connect. While the exact workings of coincidence detection in myelinated axons are unknown, synaptic plasticity and myelin plasticity might rely on the same coincidence-detection method. In this scenario, synapses would detect coincident activity and effect changes in myelination, for instance, by means of retrograde signaling to the presynaptic axon. An alternative possibility is that myelinating cells themselves might perform coincidence detection. Oligodendrocyte precursors receive direct synaptic input from neurons (Bergles et al., 2000), express NMDA receptors, the same receptors that enable coincidence detection at synapses (Káradóttir et al., 2005), and can receive inputs from multiple distant but functionally connected brain areas (Mount et al., 2019). In addition, NMDA receptors in oligodendrocyte precursors can be upregulated by brain-derived neurotrophic factor (BDNF) (Lundgaard et al., 2013), which are known to be required for myelin plasticity of premotor cortical projections (Geraghty et al., 2019). Therefore, it is also possible that myelinating cells may directly perform coincidence detection and that this process may underlie the Hebbian properties of myelin plasticity.

If myelin plasticity is Hebbian, could it also contribute to associative learning? Previous studies have described important contributions of synaptic plasticity to associative learning and memory (Tsien et al., 1996) but also highlighted that impairing synaptic plasticity does not fully abolish associative learning (Kiyama et al., 1998; Bannerman et al., 2014). Our results provide a potential explanation for these mixed findings: additional sites of plasticity may provide pathways by which Hebbian plasticity can still take place without synaptic changes. This is likely to allow associative learning and behavioral change to happen in the absence of canonical synaptic plasticity. Compatible with this hypothesis, previous studies have found effects of optogenetics-induced activity-dependent myelination on motor behavior (Gibson et al., 2014) and that learning a new motor skill leads to myelin plasticity (Sampaio-Baptista et al., 2013). Moreover, recent findings have in fact confirmed that myelin plasticity is necessary for associative learning in a Pavlovian fear-conditioning paradigm (Pan et al., 2020), which may be due to myelin plasticity’s Hebbian properties.

An intriguing possibility raised by our results is that plasticity in synapses and in myelinated axons may share broader commonalities beyond Hebb’s rule (Fields, 2015). It is now acknowledged that Hebb’s rule is part of a broader set of computational rules that regulate plasticity, such as spike-timing-dependent Plasticity (Bi and Poo, 1998) or Bienenstock-Cooper-Munro theory (Bienenstock et al., 1982). While our study only explored one spike-timing interval, which is clearly associated with Hebbian plasticity, it is possible that different spiking intervals may be associated with different types of myelin plasticity, as is the case for synaptic plasticity. For example, anti-Hebbian activity patterns are associated with decreases in synaptic strength (i.e., long-term depression). While there is still little evidence that myelin decreases can happen during healthy adulthood (Lazari et al., 2018), our results raise the question of whether anti-Hebbian stimulation could be used to induce decreases in myelination. Taken together, our results are compatible with a framework where myelin plasticity is regulated in a spike-timing-dependent manner, similar to synaptic plasticity, but further work is needed to demonstrate whether this is truly the case. Moreover, given that myelin plasticity is itself crucial in regulating spike timing during learning (Kato et al., 2020), it will be important for future work to disentangle the bidirectional interplay between spike-timing- and activity-dependent myelination.

The observation that myelin plasticity is Hebbian also provides key insights regarding what its role in brain function may be. The very existence of myelin plasticity in adulthood has been debated until recently (Purger et al., 2016; Bechler et al., 2018), as it is energetically expensive to generate the bulky macromolecules needed for forming new myelin (Harris and Attwell, 2012). While it is now established that myelin changes do happen on the scale of days to weeks (Almeida and Lyons, 2017), it still remains a mystery why the brain might need such a resource-intensive plastic process. Our results highlight that myelin plasticity may have similar computational properties to synaptic plasticity but unfold over longer timescales. This provides a role for myelin plasticity that cannot be fulfilled by synapses alone and may justify the higher energetic cost needed for the upkeep of myelin plasticity.

Beyond their role in behavior, another commonality between plasticity in synapses and myelinated axons is that they are both activity dependent. While in recent years a growing body of research has shown clear evidence that neuronal activity drives myelin changes in rodents (Gibson et al., 2014; Mitew et al., 2018; Cullen et al., 2021), our results confirm that white matter plasticity is activity dependent in humans, too. This is noteworthy not only because it proves that key findings from rodent studies can be translated to humans but also because it opens up the study of activity-dependent myelin plasticity to analyses of interindividual differences. Here, for instance, we show that interindividual differences in cortical excitability explain some variability in the induction of white matter plasticity. This hints that there may be meaningful interindividual variability in activity-driven myelin plasticity, which is unlikely to be detected in genetically and environmentally homogeneous rodent samples (Lynch and Kemp, 2014) but may be accessible in human studies. Moreover, human studies also offer the valuable possibility of combining plasticity inductions with *in vivo* functional measurements across the whole brain (Bächinger et al., 2017), which, in our case, has allowed us to describe compensatory functional changes that co-occur with white matter plasticity.

By providing evidence for activity-dependent white matter plasticity in humans, these results bridge two distinct lines of evidence on the topic. Rodent studies have largely focused on interventional, causal approaches, interrogating the activity-dependent nature of myelin plasticity (Gibson et al., 2014; Mitew et al., 2018). In contrast, human studies have focused on behavioral paradigms and their effects on white matter (Scholz et al., 2009). Our results bridge these distinct but complementary bodies of research by showing that activity-dependent white matter plasticity can be induced in humans as well and studied in conjunction with behavior. Our observations confirm that translating causal insights from rodents to humans is possible (Woolley et al., 2013; Stedehouder et al., 2019; Liu et al., 2020) and can bring about crucial discoveries on the nature and extent of brain plasticity.

Our results further build upon growing evidence that non-invasive imaging can detect subtle microstructural changes. Recent developments in MR physics are allowing researchers to measure quantitative MR parameters with higher reliability than ever before, thus providing additional tools to study the relatively subtle changes in myelination that can be experimentally induced in humans. In particular, quantitative markers based on magnetization transfer, such as the one used here, are especially sensitive to the myelin content of a voxel (Mancini et al., 2020) and have been shown to be particularly sensitive to myelin changes in response to behavioral interventions (Sampaio-Baptista et al., 2019). In summary, non-invasive quantitative markers are not only able to improve our understanding of white matter and myelin plasticity but may also afford the ability to translate key rodent findings to both healthy and clinical human cohorts (Brodt et al., 2018).

The Hebbian stimulation protocol used here, also known as paired associative TMS (or paTMS), has high translational potential. Most non-invasive brain stimulation protocols have short-lived effects of under an hour (Huang et al., 2005). This means that in clinical practice, several stimulation sessions need to be delivered over weeks to observe clinical benefits (Carpenter et al., 2012). By contrast, paTMS induces longer-lasting effects (Buch et al., 2011), which we show are still present 24 h after stimulation. This longer timescale mirrors the longer timescales of myelin plasticity (Almeida and Lyons, 2017), suggesting that protocols inducing longer-lasting effects, such as the one used here, are particularly promising candidates to induce myelin and white matter plasticity in humans. This hints that brain stimulation protocols aimed at inducing Hebbian plasticity may not only provide much-needed causal insights into basic neuroscience questions but may also be exploited for clinical use.

Using non-invasive approaches, as we do here, has the important advantage of avoiding confounding effects on glial cells from invasive plasticity inductions (Cheng et al., 2016) but poses limits to our interpretation of the results. In particular, there is extensive evidence from systematic reviews and meta-analyses linking microstructural MR signals such as MT to histological markers of myelination (Lazari and Lipp, 2021; Mancini et al., 2020). However, there is no 1-to-1 mapping between microstructural MR signals and underlying biology (Walhovd et al., 2014). Therefore, while we can infer that there are plastic changes in white matter and that these are likely driven by myelin, we cannot distinguish what exact changes in the myelinated axon are causing our observations. For instance, several types of myelin plasticity could have all happened within our experimental time frame, and given rise to our result, including (1) remodeling of existing myelin morphology (Yeung et al., 2014; Yang et al., 2020), (2) increased concentration of existing myelin due to shortening of nodes of Ranvier (Arancibia-Carcamo et al., 2017), (3) existing oligodendrocytes producing new myelin around unmyelinated axons (Bacmeister et al., 2020; Steadman et al., 2019; Hughes et al., 2018), and (4) existing oligodendrocytes producing new myelin around available regions of discontinuously myelinated axons (Young et al., 2013; Tomassy et al., 2014; Hughes et al., 2018; Swire et al., 2019; Bacmeister et al., 2020). It is also a possibility that *de novo* oligodendrogenesis may have taken place over 24 h, as production of myelin by new oligodendrocytes is known to take place over a restricted time window ranging between 2 h and 4 days (Czopka et al., 2013; Xiao et al., 2016; Bacmeister et al., 2020). Moreover, axonal plasticity and myelin plasticity are often interlinked (Sinclair et al., 2017; Ford et al., 2015; Almeida and Lyons, 2017), but our results cannot assess the contribution of plasticity in axonal diameter to the overall changes in myelination that we observed. In summary, a large variety of candidate processes have been proposed to contribute to plasticity of the myelinated axon (Almeida and Lyons, 2017; Kaller et al., 2017), and while any of them could be driving our observations, our results hint that at least some of them are bound to be Hebbian in nature.

### Conclusions

In conclusion, our study combines recent advances in non-invasive brain imaging and brain stimulation to show that Hebb’s rule extends beyond synapses. While myelin plasticity may provide an additional site of brain plasticity, the same rules may constrain its functions. As our understanding of non-synaptic forms of brain plasticity develops, we suggest that Hebb’s rule may be a broader principle than previously thought, constraining multiple plastic processes in the human brain.

### Limitations of the study

The study also presents several limitations, particularly in relation to the establishment of causality in our observations. Non-invasive brain stimulation provides crucial opportunities to draw causal inference in humans, and a key strength of our TMS-based approach is that we have been able to establish a causal link between coordinated neuronal activity and myelin changes. However, experimenting in humans means that it is challenging to disentangle the processes that mediate this link. A key confounding factor is that Hebbian stimulation is known to also induce synaptic plasticity, and it is difficult to disentangle the relative contributions of synaptic and myelin plasticity to the changes we observed in behavior and in functional connectivity. For example, it is possible that rapid synaptic plasticity in the connections between PMv and M1 may have contributed to the establishment or consolidation of myelin plasticity. Indeed, cortical excitability of M1 is sensitive to changes in synaptic strength in the pathway connecting PMv to M1 (Gerschlager et al., 2001; Paus et al., 2001), and it is challenging to distinguish whether changes in myelination have driven the observed increases in cortical excitability, or vice versa. In summary, while we can be confident that coordinated neuronal activity played a causal role in inducing myelin changes, further studies in non-human samples may be needed to dissect the precise pathways underlying this causal link.

An additional limitation is that our study only explored myelin plasticity at one time point: 24 h after Hebbian stimulation. While myelin produced by Hebbian stimulation is likely still present a few days after the stimulation paradigm (Gibson et al., 2014), we did not test for the persistence of white matter or behavioral changes beyond 24 h in the current study. Our experimental approach in humans may not be best placed to address this question, given the wide variety of experience that human participants will have over days to weeks, the effects of which might mask subtle effects induced by the experimental manipulation. Nonetheless, how long experience- and activity-dependent myelin changes persist in the adult brain is still an open question, and further work is needed to better understand the timescales of activity-dependent myelin changes.

## STAR★Methods

### Key resources table

**Table:** 

REAGENT or RESOURCE	SOURCE	IDENTIFIER
**Deposited data**		

Statistical maps and Neuroanatomical masks	This paper	https://doi.org/10.5281/zenodo.6532370

**Software and algorithms**		

Matlab 2018b	MathWorks	N/A
Brainsight (version 2.3.12)	Rogue Resolutions Ltd	N/A
Picoscope 6 (version 2.3.12)	PicoTech	N/A
FMRIB Software Library (FSL) v6.0	Wellcome Centre for Integrative Neuroimaging, FMRIB, University of Oxford	N/A
Deposited Software	This paper	https://doi.org/10.5281/zenodo.6532370

**Other**		

Hum Bug 50/60 Hz Noise Eliminator	Quest Scientific	N/A
CED power1401	Cambridge Electronic Design Limited	N/A
PicoScope	PicoTech	N/A
DuoMAG MP-Dual TMS monophasic stimulators	DeyMed DuoMag, Rogue Resolutions Ltd	N/A
D440 Isolated EMG amplifier	Digitimer	N/A

### Resource availability

#### Lead contact

Further information and requests for resources should be directed to and will be fulfilled by the Lead Contact, Alberto Lazari (alberto.lazari@ndcn.ox.ac.uk).

#### Materials availability

This study did not generate new unique reagents.

### Experimental model and subject details

#### Experimental design of study 1 and study 2

All participants underwent three consecutive days of testing (Figure 1A). On the first day, Magnetic Resonance Imaging (MRI) was collected (including myelin markers). On the second day, the participants underwent either Hebbian or non-Hebbian plasticity induction (both achieved through Transcranial Magnetic Stimulation, TMS). On the third day, MRI (including myelin markers) was collected again. Each participant’s sessions were matched to be at the same time of day to control for circadian effects. All participants were self-assessed right-handed and their handedness was further confirmed through the Edinburgh Handedness Inventory (Oldfield, 1971). All participants were screened for TMS and MRI safety, received monetary compensation for their participation, and gave their informed consent to participate in this study. All study procedures were reviewed and approved by the local ethics committee at the University of Oxford (Central University Research Ethics Committee (CUREC)), and followed the Declaration of Helsinki.

In study 1, 19 healthy participants (aged 18–32, 9 female) underwent a longitudinal MRI-TMS-MRI paradigm, and all participants underwent the Hebbian plasticity-induction condition.

In study 2, 36 healthy participants (aged 19–30, 22 female) underwent a longitudinal MRI-TMS-MRI paradigm. Participants were randomly assigned either to the Hebbian or the non-Hebbian plasticity induction protocols.

### Method details

#### Hebbian and non-hebbian plasticity induction protocols

Hebbian and non-Hebbian protocols were both based on paired associative cortio-cortical Transcranial Magnetic Stimulation (paTMS), a recently developed stimulation protocol (Rizzo et al., 2009, 2011; Koganemaru et al., 2009; Buch et al., 2011; Johnen et al., 2015) where two cortical regions are repetitively stimulated in a paired fashion at inter-pulse intervals known to induce LTP-like associative synaptic plasticity.

Hebbian (active) and non-Hebbian (control) stimulation protocols both used two DuoMAG MP-Dual TMS monophasic stimulators (DeyMed DuoMag, Rogue Resolutions Ltd.) to deliver paired pulses via two figure-eight coils, one 70mm-diameter coil over primary motor cortex (M1) and one 50mm-diameter coil over ventral premotor cortex (PMv). In the Hebbian condition, the paired pulses were 6ms apart, mimicking the timing of synaptic plasticity inductions used *in vitro*. In the non-Hebbian condition, the pulses were 500 ms apart, which is long enough to avoid physiological interactions between the two pulses which may take place at shorter intervals (Valls-Solé et al., 1992). Moreover, using a 500 ms interval has been shown not to have behavioural and physiological effects in previous studies (Johnen et al., 2015). All other parameters were the same across protocols: both protocols consisted of 90 paired TMS pulses, delivered at 0.1 Hz over a 15 min period, without interruptions. For both protocols, the M1 coil was set at a SI_1mV_ intensity, whereas the ventral premotor cortex coil was set at a 110% resting Motor Threshold intensity (rMT). SI_1mV_ was determined as the intensity giving reliable and stable 1 mV Motor-Evoked Potentials (MEPs) at rest over 10 pulses. rMT was determined as the intensity at which 5 out of 10 pulses gave no MEP response greater than 0.05 mV.

Both protocols were performed at rest, with the participant resting their hands on a pillow and watching a series of still images on a computer screen. In summary, each area, PMv and M1, was stimulated in an identical manner in the two protocols; each was stimulated the same number of times at the same intensity and frequency and for the same duration as in the other protocol, but the relative timing of stimulation meant that spike-timing-dependent plasticity could only occur in one protocol.

Subjects were blind to their experimental condition throughout the experiment. Experimenters were also blind to the experimental condition prior to stimulation; however, the subtle difference in stimulation timing between Hebbian stimulation and non-Hebbian stimulation made it impossible to achieve full blinding once the stimulation had started taking place. At the end of the experiment, participants were administered a discomfort questionnaire (Meteyard and Holmes, 2018) and a questionnaire aimed at assessing blinding of the experiment (both available here: https://open.win.ox.ac.uk/pages/alazari/hebbian-white-matter-plasticity/). The scores from the blinding questionnaire were used to calculate an overall Bang’s Blinding Index for the experiment (Bang et al., 2004).

#### Neuronavigation

All stimulation was delivered using continuous tracking of coil location with respect to subject neuroanatomy (i.e. neuronavigation). This was achieved through a Polaris camera and the Brainsight software (Rogue Resolutions, Inc.), and used the participant’s T1-weighted (T1w) structural MRI scan. The participant was tracked via a headband with reflective spheres attached to it; the coils were tracked with coil trackers that were re-calibrated at the beginning of each testing day. Online neuronavigation ensured that all stimulation sites were within 3 mm of target location, as described in previous publications (Buch et al., 2011).

Coil location was also recorded and analysed offline. An automated Brainsight tool was used to find the closest brain voxel to the sampled stimulation site. The coordinates for this voxel were then transformed to standard space to allow overlaying of stimulation sites from different participants. At this stage, a total of 42 stimulation locations were included, as 4 participants’ stimulation locations failed to save due to software fault (2 in active-only study, 1 in active randomised and 1 in control randomised), and 5 participant’s stimulation locations could not be automatically determined with Brainsight (2 in active-only, 2 in active randomised, 1 in control randomised). Because the magnetic field may reach 30% of its peak level throughout a region with a diameter of 4 cm (Siebner et al., 2009), spheres of 4 cm diameter were created around the sample stimulation location to provide a conservative estimate of the spatial specificity achieved by TMS. These spheres were then overlaid upon each other. All stimulation sites were within 3 mm of target location, as described in previous publications (Buch et al., 2011).

#### Cortical physiology

As a measure of cortical excitability, we determined the Stimulator Intensity giving reliable and stable 1 mV Motor-Evoked Potentials in the First Dorsal Interosseus muscle of the right hand (‘SI_1mV_’) (Stefan et al., 2002). The SI_1mV_ value was determined at rest and based on 10 TMS pulses. This measure was collected before Hebbian stimulation on day 2 and before MRI scanning on day 3, taking care that sessions were matched to be at the same time of day to control for circadian effects. The SI_1mV_ value was collected in an exploratory manner in the last 7 participants of study 1, and in all participants of study 2 to confirm the presence of longitudinal effects.

#### Magnetic resonance imaging of myelin

Participants underwent Magnetic Resonance Imaging (MRI) sessions 24 h before and 24 h after the plasticity induction protocol. MRI data were collected with a 3.0-T Prisma Magnetom Siemens scanner, software version VE11C (Siemens Medical Systems, Erlangen, Germany). T1-weighted structural imaging (T1w), Diffusion-Weighted Imaging (DWI) and Multi-Parameter Mapping (MPM) sequences were collected.

The T1w sequence (TR = 1900 ms, TE = 3.96 ms, resolution = 1 mm isotropic, GRAPPA = 2) had a large Field of View (FOV = 256 mm^3^)to allow for the nose and intertragic notches of the ears to be included in the image to facilitate later neuronavigation of the TMS coil to the target position.

Diffusion-weighted Echo-planar imaging (EPI) scans (TR = 3070 ms, TE = 85.00 ms, FOV = 204 mm^3^, resolution = 1.5 mm isotropic, multiband factor of 4) were collected for two b-values (500 and 2000 s/mm^2^), over 281 directions. An additional 23 volumes were acquired at b = 0, 15 in anterior-posterior (AP) phase-encoding direction and 8 in the posterior-anterior (PA) phase-encoding direction.

The MPM protocol (Weiskopf et al., 2013) included three multi-echo 3D FLASH (fast low-angle shot) scans with varying acquisition parameters, one RF transmit field map (B1+map) and one static magnetic (B0) field map scan, for a total acquisition time of roughly 22 min. To correct for inter-scan motion, position-specific receive coil sensitivity field maps, matched in FOV to the MPM scans, were calculated and corrected for (Papp et al., 2016). The three 3D FLASH scans were designed to be predominantly T1-, PD-, or MT-weighted by changing the flip angle and the presence of a pre-pulse: 8 echoes were predominantly Proton Density-weighted (TR = 25 ms; flip angle = 6 degrees; TE = 2.3–18.4 ms), 8 echoes were predominantly T1-weighted (TR = 25 ms; flip angle = 21 degrees; TE = 2.3–18.4 ms) and 6 echoes were predominantly Magnetisation Transfer-weighted (MTw, TR = 25ms; flip angle = 21 degrees; TE = 2.3–13.8 ms). For MTw scans, excitation was preceded by off-resonance Gaussian MT pulse of 4 ms duration, nominal flip angle, 2 kHz frequency offset from water resonance. All FLASH scans had 1 mm isotropic resolution, field of view (FOV) of 256 × 224 × 176 mm^3^, and GRAPPA factor of 2 × 2. The B1 map was acquired through an EPI-based sequence featuring spin and stimulated echoes (SE and STE) with 11 nominal flip angles, FOV of 192 × 192 × 256 mm^3^ and TR of 500 ms. The TE was 37.06 ms, and the mixing time was 33.8 ms. The B0 map was acquired to correct the B1+ map for distortions due to off-resonance effects. The B0 map sequence had a TR of 1020.0 ms, first TE of 10 ms, second TE of 12.46 ms, field of view (FOV) of 192 × 192 × 256 mm^3^ and read-out bandwidth of 260 Hz/pixel.

MRI scan pre-processing, analysis and statistical comparisons were performed using FMRIB Software Library (FSL, v6.0) (Smith et al., 2004), except for the MPM quantitative map estimation step which was carried out using the hMRI toolbox implemented in Matlab-based SPM, as described in (Tabelow et al., 2019). All T1w images were preprocessed through a standard FreeSurfer-based pipeline (Fischl et al., 2004; Glasser et al., 2013) to correct for bias field and achieve ACPC alignment (for use in Neuronavigation). For longitudinal analyses of MRI, a midpoint T1w space was derived as done in previous studies (Scholz et al., 2009).

Custom pipelines based on existing FSL tools were developed to preprocess diffusion and Magnetisation Transfer saturation (MT) data (code available here: https://open.win.ox.ac.uk/pages/alazari/hebbian-white-matter-plasticity/). For diffusion, the *topup* tool was run on average images of AP b0 volumes and PA b0 volumes. The resulting susceptibility-induced off-resonance field was then used as an input for the eddy tool (Andersson and Sotiropoulos, 2016), which was run with options optimised for multiband diffusion data to correct for eddy currents and subject movement.

Magnetisation Transfer saturation (MT) quantitative maps were estimated through the hMRI toolbox (Tabelow et al., 2019). MPM volumes were then registered to Montreal Neurological Institute (MNI) space by combining the registration between MPM volumes and midpoint T1w images with the registration between the midpoint T1w space and the MNI template; these volumes were then smoothed with a Gaussian kernel of 3 mm. At this stage, 1 participant was excluded as their MPM scans were heavily corrupted due to movement artefacts (study 1); 1 participant was excluded due to lower quality signal in the MPM scans, which resulted in poor registration to template (study 2, control condition); 1 participant was excluded due to a slight callosal abnormality preventing registration to template (study 2, active condition).

#### Magnetic Resonance Imaging of resting-state connectivity

The rs-fMRI Echo-planar imaging (EPI) sequence (TR = 750 ms; TE = 29.00 ms, resolution = 2 mm isotropic, FOV = 208 mm^3^) employed fat saturation-based fat-suppression, had a multiband factor 6 and used GRAPPA with acceleration factor 2. Participants were asked to keep their eyes open and let their mind wander during this sequence. The screen was kept black for the duration of this scan, and an eye tracker was used to ensure the participant was awake. rs-fMRI data was preprocessed with high pass filter cutoff of 100 s, MCFLIRT to correct for motion, smoothing at sigma of 3 mm, BBR registration, and fieldmap-based B0 unwarping. Single session Multivariate Exploratory Linear Optimized Decomposition into Independent Components (MELODIC)-based Independent Component Analysis was used to extract components at the single subject level. Components were classified as noise or signal manually for the first few subjects, and the labels were then used to build a FIX-based classifier to denoise the data (Griffanti et al., 2017). Finally, dual regression (Beckmann et al., 2009) was used to estimate connectivity maps from the stimulated ROIs.

### Quantification and statistical analysis

#### Statistical inference for magnetic resonance imaging data

Group-level analyses of MT and rs-fMRI maps were conducted through nonparametric permutation inference in the *randomise* tool (Winkler et al., 2014), controlling for the family-wise error rate. Change maps were calculated for each subject by subtracting the day 3 map from the day 1 map. Analyses were run in MNI space, with 10,000 permutations and Threshold-Free Cluster Enhanced (Smith and Nichols, 2009). Membership of different studies (Study 1 and Study 2) was encoded as a covariate, to allow for contrasts to test whether effects were present in each study. Changes in the SI_1mV_ metric of cortical physiology (see above) were used as the regressor of interest, explicitly testing for interactions between Hebbian and non-Hebbian conditions. In a separate model, group-level differences between Hebbian and non-Hebbian conditions were also tested through an unpaired t-test.

#### Reconstruction of stimulated fiber bundles

Using Diffusion-Weighted Imaging (DWI) data, we reconstructed the white matter fiber bundles stimulated in our plasticity induction protocols. White matter bundles connecting the stimulated cortical sites were estimated using multi-fibre probabilistic diffusion tractography through Probtrackx (Behrens et al., 2007). Regions of interest (ROI) in the cortex were based on the neuronavigation-derived sites for each participant, as described above. As the motor hotspot does not always overlap with the postcentral gyral fold, and a larger coil was used for M1 compared to PMv, the motor hotspot ROI was enlarged to a 3cm radius to improve the output tract quality. Tractography was run in native DWI space, with outputs in Montreal Neurological Institute (MNI) space to enable pooling of results across all subjects. Individual-level maps of streamline densities were thresholded at 1% of the number of total valid streamlines per subject, binarised, and then overlaid.

#### Action reprogramming task

Participants completed the Action Reprogramming task before and immediately after plasticity induction. The Action Reprogramming task aimed to probe action execution and action reprogramming (Isoda and Hikosaka, 2007; Neubert et al., 2010). Cues consisted of a central square (either red or green) with two ‘flanker’ squares (one red and one green). Participants were instructed to press the button on the side where the flanker colour matched the colour of the central square. The flankers kept switching sides at random, whereas the central square was the same colour for 3–7 trials at the time (Figure 3). This way, participants simply had to execute a movement when the central cue colour stayed the same (‘stay trials’), but they had to inhibit the movement and carry out a different one in the trials where the central cue colour had switched (‘switch trials’). Each participant underwent a session of 112 switch trials and corresponding stay trials (for a total of 678 trials). Participants were told to be as fast and accurate as they could. They received detailed task instructions in paper format at the beginning of each session; in addition, the instructions were reiterated in a computer-based fashion at the beginning of the baseline task (code available here: https://open.win.ox.ac.uk/pages/alazari/hebbian-white-matter-plasticity/). Before the baseline task, they undertook roughly 100 trials to make sure first that they understood the rules of the task, and that they had habituated to the task. Three participants (1 in study 2, control condition; 2 in study 2, active condition) had recently performed the same action reprogramming task extensively as part of a separate experiment, and were thus excluded from behavioural analyses to avoid the possibility of training and/or carry-over effects.

#### Action reprogramming meta-analysis

A meta-analysis of task-based neuroimaging studies involving action reprogramming was run using the NeuroQuery tool (Dockès et al., 2020). NeuroQuery performs multivariate prediction-based meta-analyses using text-based search terms and produces meta-analytic activation maps that refer to the concept of interest. As the previous literature consistently refers to our concept of interest as ’action reprogramming’ (Neubert et al., 2010), we used this as the search term for our meta-analysis.

#### Statistical inference for cortical physiology and action reprogramming data

Analyses of cortical physiology (with SI_1mV_ as the key variable of interest) and Action Reprogramming behaviour (with Reaction Times as the key variables of interest) were run in GraphPad Prism (GraphPad Software, La Jolla, California, US) with the exception of the ANCOVA analysis of Reaction Times which was run in SPSS (SPSS Statistics, IBM Corp.). Longitudinal analyses across all groups were run as one-way ANOVAs of longitudinal change scores, with Dunn’s multiple comparison tests as post-hoc tests. Longitudinal analyses across all groups which aimed to covary for additional factors were run as ANCOVAs. Alpha level for statistical significance was set at 0.05 and all Confidence Intervals (CIs) were set at 95% confidence.

## Data Availability

• Raw data reported in this paper will be shared by the lead contact upon request. • All original code has been deposited here: (https://open.win.ox.ac.uk/pages/alazari/hebbian-white-matter-plasticity/, https://doi.org/10.5281/zenodo.6532370) and is publicly available as of the date of publication. DOIs are listed in the key resources table. • Any additional information required to reanalyze the data reported in this paper is available from the lead contact upon request.

## Supplemental information

Document S1. Figures S1–S4

Document S2. Article plus supplemental information
