# Supplementary material for: Hebbian activity-dependent plasticity in white matter
Source: Cell Rep. 2022 Jun 14;39(11):110951. doi: 10.1016/j.celrep.2022.110951 (PMC9376741; doi:10.1016/j.celrep.2022.110951)
Supplement: Document S1. Figures S1–S4 [file mmc1.pdf]

Cell Reports, Volume *39*

Supplemental information

Hebbian activity-dependent plasticity in white matter

Alberto Lazari, Piergiorgio Salvan, Michiel Cottaar, Daniel Papp, Matthew F.S. Rushworth, and Heidi Johansen-Berg

Supplemental Results

A

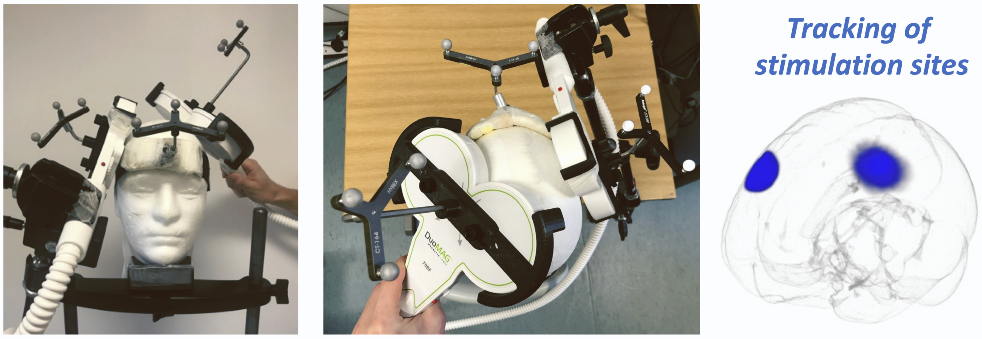

B

Blinding and matching of conditions in Study 2

	**Active (*n=18*)**		**Control (*n=18*)**	
	Mean	*SD*	*Mean*	*SD*
Age	25.167	3.240	23.722	2.824
Handedness	88.529	13.076	86.013	15.109
Distraction	4.056	2.235	5.333	1.847
Annoyance	3.722	2.347	3.944	2.100
Head twitches	2.389	2.279	1.667	2.196
Face twitches	1.000	1.715	1.278	1.904
**Bang's Blinding index**	**0.111**	**0.458**	**0.000**	**0.460**

	**Active**	**Control**
	# participants	*# participants*
**Reported other side effects**	3	1
**Gender**		
female	12	10
male	6	8
**Time of day**		
morning	6	11
afternoon	12	7

No difference in anatomical targeting across conditions

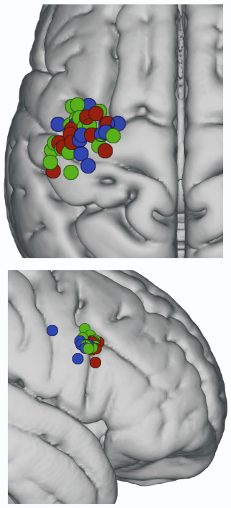

Supplemental Figure 1: **Details of Neuronavigation set-up, Randomisation and Blinding.** A. Neuronavigation set-up. *Left*: All stimulation was delivered using continuous tracking of coil location with respect to subject neuroanatomy (i.e. neuronavigation), which was achieved through reflective sphere attached to headbands and coil holders. Online neuronavigation ensured that all stimulation sites were within 3 mm of target location. *Right*: Coil location was also recorded and used for further analyses offline. Here, the stimulation location for all subjects are overlaid in a single 3D image. B. Randomisation and Blinding. *Left*: In Study 2, participants in the Hebbian (active) and Non-Hebbian (control) groups were well matched for demographic variables such as age and gender. Furthermore, the two stimulation protocols did not lead to different experiences of stimulation side-effects, and blinding was successful in both stimulation conditions. *Right*: Offline analysis of neuronavigation target locations shows similarity in the anatomical targeting of stimulation across participants in Study 1 and Study 2, independently of the assigned condition. PMv stimulation was targeted in an anterior position on the boundary between ventral area 6 and area 44 (Tomassini et al. 2007, Neubert et al. 2014) adjacent to the inferior precentral sulcus. All panels are related to Figure 1.

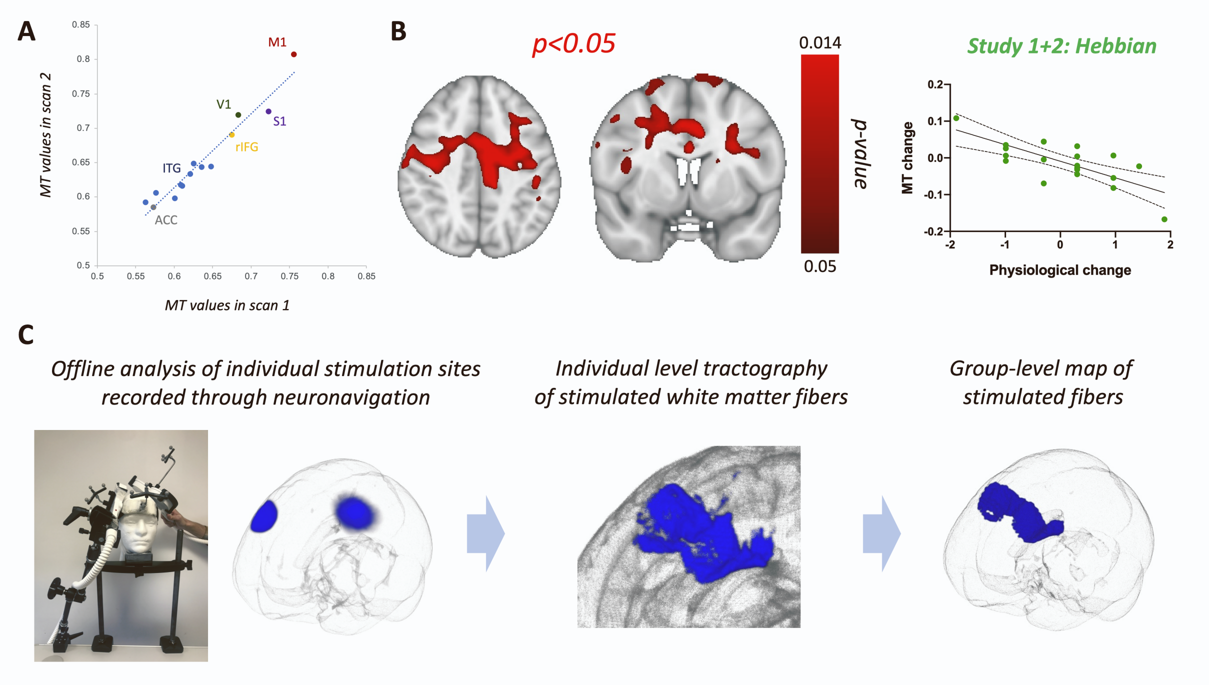

Supplemental Figure 2: **Microstructural Imaging and Tractography of stimulated white matted fibres. A. MT has high test-retest reliability on the same scanner across different days.** One subject (author A.L.) underwent MPM scanning on the same scanner on different weekdays. Values from MT maps across a range of ROIs have high test-retest reliability within the same scanner. **B. Microstructural results in Hebbian subjects only.** To further confirm the correlation between physiological change and MT change, we ran a voxelwise analysis on MT using physiological change as a regressor in Hebbian subjects only (across Study 1 and Study 2). This generates a similar cluster of significant correlation, extending across both hemispheres (A), where greater increases in excitability (more negative physiological change score) are associated with greater increases in MT. This confirms that physiological change is correlated with MT change in white matter, even when considering the Hebbian group alone without contrasting this correlation with the one in the control group. **C. Tractography of stimulated white matter fibres.** *Left*: Neuronavigation allows recording of exact sitmulation locations for each subject. *Centre*: Using stimulation locations for each individual, we estimate individual-level white matter fibers stimulated in our paradigm. *Right*: The group-level map of tract overlap across subjects is shown in blue. All panels are related to Figure 2.

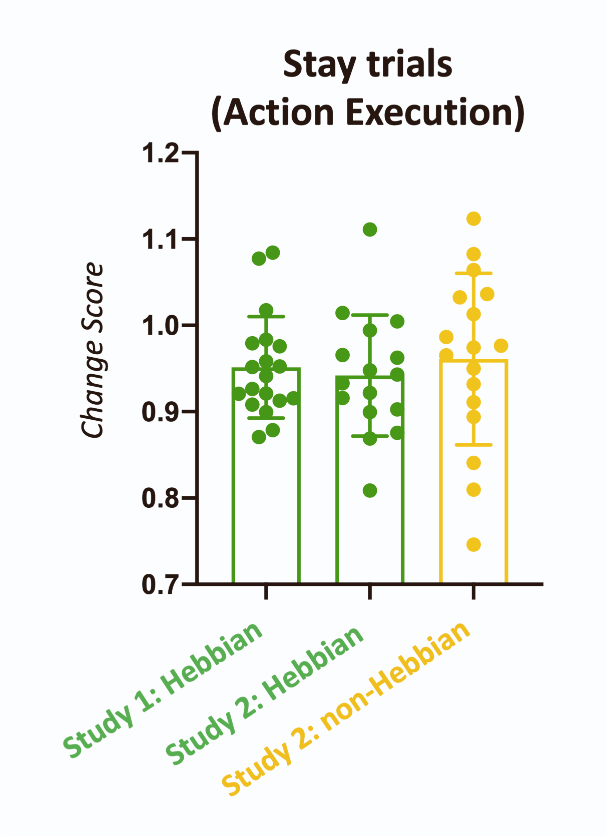

Supplemental Figure 3: **Behavioural effects of Hebbian stimulation do not extend to action execution.** Each dot in the graph represents the change in stay trial Reaction Time for one subject. When considering stay trials (action execution), no significant difference was found between groups (one-way ANOVA effect of group: $F(2, 51)=1.100$, $p=0.5769$). This figure relates to Figure 3.

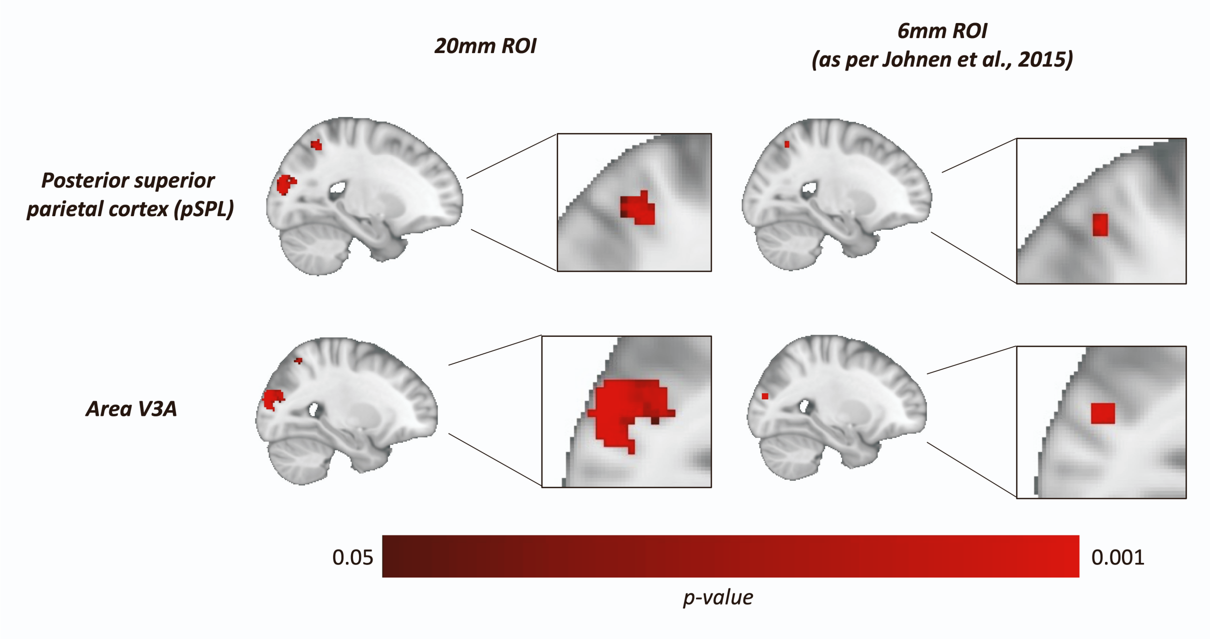

Supplemental Figure 4: **Large-scale compensatory changes in resting-state connectivity, explored through ROI analyses.** Related to Figure 4.
